# Dynamics of the digestive acquisition of bacterial carriage and integron presence by French preterm newborns according to maternal colonization: The DAIR3N multicentric study

**DOI:** 10.3389/fmicb.2023.1148319

**Published:** 2023-03-14

**Authors:** Alice Patry, Philippe Bothorel, Anaïs Labrunie, Laurent Renesme, Philippe Lehours, Melinda Benard, Damien Dubois, Laure Ponthier, Sylvain Meyer, Karine Norbert, Laurent Villeneuve, Philippe Jouvencel, David Leysenne, Delphine Chainier, Sandrine Luce, Carole Grélaud, Marie-Cecile Ploy, Antoine Bedu, Fabien Garnier

**Affiliations:** ^1^INSERM UMR, Limoges University, Limoges University Hospital, Limoges, France; ^2^Department of Pediatrics, Mother-Child Hospital, Limoges University Hospital, Limoges, France; ^3^Epidemiology, Biostatistics, and Research Methodology Centre (CEBIMER), Limoges University Hospital, Limoges, France; ^4^Department of Pediatrics, Neonatology and Maternity Unit, Pellegrin University Hospital, Bordeaux, France; ^5^Bacteriology Laboratory, Pellegrin University Hospital, Bordeaux, France; ^6^Department of Pediatrics and Neonatology, CHU Toulouse, Toulouse, France; ^7^Bacteriology and Hygiene Department, Federative Institute of Biology, CHU Toulouse University Hospital, Toulouse, France; ^8^Department of Pediatrics, Pau Hospital, Pau, France; ^9^Medical Biology Laboratory, Pau Hospital, Pau, France; ^10^Department of Pediatrics and Neonatology, « Côte Basque » Hospital, Bayonne, France; ^11^Microbiology Laboratory, « Côte Basque » Hospital, Bayonne, France

**Keywords:** Integrons, digestive acquisition, antimicrobial resistance, preterm newborn infant, resistance markers, gram-negative bacteria

## Abstract

**Objectives:**

The study aimed to describe the dynamics and risk factors of Gram-negative bacteria (GNB) acquisition in preterm infants.

**Methods:**

This prospective multicenter French study included mothers hospitalized for preterm delivery and their newborns, followed until hospital discharge. Maternal feces and vaginal fluids at delivery, and neonatal feces from birth to discharge were tested for cultivable GNB, potential acquired resistance, and integrons. The primary outcome was the acquisition of GNB and integrons in neonatal feces, and their dynamics, evaluated by survival analysis using the actuarial method. Risk factors were analyzed using Cox models.

**Results:**

Two hundred thirty-eight evaluable preterm dyads were included by five different centers over 16 months. GNB were isolated in 32.6% of vaginal samples, with 15.4% of strains producing extended-spectrum beta-lactamase (ESBL) or hyperproducing cephalosporinase (HCase), and in 96.2% of maternal feces, with 7.8% ESBL-GNB or HCase-GNB. Integrons were detected in 40.2% of feces and 10.6% of GNB strains. The mean (SD) length of stay of newborns was 39.5 (15.9) days; 4 died in the hospital. At least one infection episode occurred in 36.1% of newborns. The acquisition of GNB and integrons was progressive from birth to discharge. At discharge, half of newborns had ESBL-GNB or HCase-GNB, independently favored by a premature rupture of membranes (Hazard Ratio (HR), 3.41, 95% confidence interval (CI), 1.71; 6.81), and 25.6% had integrons (protective factor: multiple gestation, HR, 0.367, 95% CI, 0.195; 0.693).

**Conclusion:**

In preterm newborns, the acquisitions of GNB, including resistant ones, and integrons are progressive from birth to discharge. A premature rupture of membranes favored the colonization by ESBL-GNB or Hcase-GNB.

## Introduction

Neonatal sepsis, especially in preterm infants (Oza et al., 2015; Cortese et al., 2016; Thomson et al., 2021) and when multidrug-resistant bacteria (MDRBs) are involved (Laxminarayan et al., 2016; Yusef et al., 2018), is associated with a high mortality and morbidity burden. Control of the spread of MDRBs and research into the emergence of MDRBs in pediatrics are priorities for the World Health Organization (WHO; World Health Organization, 2001).

Antibacterial resistance can occur through mutation or acquisition of resistance genes by horizontal transfer of mobile genetic elements (Martinez, 2014). Although the frequency of horizontal gene transfer in the human microbiome remains controversial, the human microbiome is now recognized as a reservoir of antibacterial resistance genes (Brinkac et al., 2017; Ruppé et al., 2019). Colonization of the infant’s digestive tract occurs during and after birth (Schoenmakers et al., 2019). Colonization with MDRBs may occur by transmission during birth or from the neonatal environment during the first weeks of life (Basu, 2015; Barraud et al., 2018). Intestinal colonization by Gram-negative bacteria (GNB) may cause sepsis in preterm newborns, mainly through translocation (Basu, 2015).

Integrons are genetic elements in bacteria capable of capturing, exchanging, and expressing genes embedded in gene cassettes arrays (Cambray et al., 2010). Together with plasmids and transposons, they play an important role in the acquisition of antibacterial resistance (Ravi et al., 2015). Different classes are defined according to the sequence of their associated integrase. Classes 1, 2 and 3 are involved in antibacterial resistance, with class 1 integrons being the most prevalent in clinical settings (Cambray et al., 2010). The association between the presence of integrons and multidrug resistance in *Enterobacterales* has been demonstrated in several studies (Leverstein-van Hall et al., 2003; Nijssen et al., 2005; Daikos et al., 2007). The absence of integrons may be associated with the absence of acquired resistance to third generation cephalosporins (3GC), aminoglycosides and fluoroquinolones (Barraud et al., 2014; Mendes Moreira et al., 2019). Recently, two studies showed a good negative predictive value of class 1 and class 2 integrons for the presence of resistance to 3GC, aminoglycosides, and trimethoprim/sulfamethoxazole (SXT) (Barraud et al., 2021; Elias et al., 2022). Thus, integrons are an important means of spreading resistance to antibiotics and could be used as resistance markers.

A previous study conducted by our team in term newborns showed that GNB and integrons in the neonatal intestinal tract are mostly acquired after birth, and that the antibiotics administration during delivery is a major risk for integrons acquisition (Barraud et al., 2018). Here, we wanted to investigate the dynamic of the acquisition of GNB and integrons in preterm newborns, considering the fecal and vaginal colonization of the mothers. The production of extended-spectrum beta-lactamase (ESBL) or hyperproduced cephalosporinase (Hcase) was used as a probe for multi-resistance to antibiotics. For sake of simplicity, we named the GNB strains harboring either of these two phenotypes of beta-lactam resistance as MDR-GNB. Risk factors associated with the GNB acquisition were evaluated. In addition, data in newborns were analyzed considering the carriage of GNB and integrons in the vaginal and fecal samples of the dyad’s mother collected at the time of birth.

## Patients and methods

### Study design

This observational prospective multicenter study, called DAIR3N, was conducted from 2014 to 2016 in tertiary neonatology units of five cities in the South-West of France: Bayonne, Bordeaux, Limoges, Pau and Toulouse.

### Study population and study period

Study participants were all consecutive mothers and their infants who were hospitalized at participating centers for preterm birth at a gestational age between 28 and 34 weeks or with a birth weight of 1,500 g or less and with a hospitalization in the neonatal unit expected to last at least 3 weeks. Newborns were excluded if they were born outside the participating centers, or if they had congenital digestive defects.

### Ethics statement

The DAIR3N study was approved by the Ethic Committee of Limoges (*Comité pour la Protection des Personnes du Sud-Ouest et Outremer #4*) on 18 March 2014 (Approval number #CPP14-017/2014-A00283-44). Mothers signed informed consent prior to the inclusion of the dyad.

### Clinical data

The main clinical data collected with regards to the mother were her age, number of pregnancies, whether the pregnancy was multiple or not, and, during the pregnancy, the carriage of *Streptococcus agalactiae*, infection diagnosis, and antibiotic exposure. Main data on delivery comprised the etiology of premature delivery if available, mode of delivery, a maternal fever, antibiotic exposure during delivery, and the duration of the membranes rupture. With regards to newborns, the main data collected were sex, weight at birth and discharge, the feeding mode, whether an infectious episode was suspected or confirmed, and whether antibiotics were administered.

### Samples collection and processing

In mothers, a swab of vaginal fluids before delivery and of feces after delivery were collected as much as possible. For newborns, meconium samples were collected at birth, then feces were collected at day 7, every two weeks until discharge, and at discharge.

Samples were inoculated onto Drigalski agar plates (BioMérieux, Marcy l’étoile, France) and incubated at 35–37°C for 48 h, as performed for the previous study (Barraud et al., 2018). All samples were stored at −80°C in cryotubes for further integron detection directly in the sample. According to the center, GNB isolates with various morphotypes were identified by a MALDI-TOF mass spectrometry system (bioMérieux, Marcy l’étoile, France or Brucker, Palaiseau, France) or a Vitek 2^®^ system using the ID-GN cards (BioMérieux, Marcy l’étoile, France) as recommended by the manufacturer. Antibiotic susceptibility testing was performed using a Vitek 2^®^ system (bioMérieux, Marcy l’étoile, France) or a Phoenix^®^ system (Becton Dickinson, Le Pont-de-Claix, France) or by diffusion on agar plates, according to the EUCAST-SFM recommendations (Eucast Jehl et al., 2014). All GNB were then stored at −80°C. Antibiotics susceptibility results were analyzed as recommended by the CA-SFM looking for different types of acquired resistance: (i) extended-spectrum beta-lactamase (ESBL) and hyperproduced cephalosporinase (Hcase), both identified as markers of multidrug resistance, and (ii) all other types of acquired resistance, such as resistance to beta-lactams due to beta-lactamases other than ESBL or Hcase, to quinolones, to aminoglycosides, or to SXT.

Total DNA from samples was extracted using the NucliSens^®^ easyMAG extractor (bioMérieux, Marcy l’étoile, France) as recommended by the manufacturer. For the integron detection, a multiplex real-time PCR enable to detect class 1, 2, and 3 integrons was performed on each DNA extract and on each GNB using a MX3005P^®^ system (Stratagene, San Diego, California, United States) as previously described (Barraud et al., 2010). Strains belonging to the same bacterial species isolated both from the newborn fecal samples and from the mother fecal and/or vaginal samples were compared by means of random amplified polymorphic DNA (RAPD) using primer ERIC-2 as described (Meacham et al., 2003).

### Main outcomes

The primary outcome was the acquisition of GNB and/or integrons in neonatal feces, and its dynamics. The risk factors for the acquisition of GNB, MDR-GNB and integrons were evaluated.

### Statistical methods

The statistical analyses were conducted with SAS Enterprise Guide software (SAS Institute Cary, NC). Analyses were conducted on dyads, i.e., the pairs formed by each newborn and his/her mother. The primary endpoint, the dynamics of the acquisition of GNB and/or integrons in the feces of newborns, was evaluated according to a survival analysis using the actuarial method. The risk factors associated with the acquisition of GNB, of MDR-BGN, of other acquired resistance and of integrons were first analyzed by univariate analysis using a Cox model for interval-censored data on the potential factors (Finkelstein, 1986). This model was then implemented for multivariate models with factors whose significance in univariate analysis was less than 0.20. For all tests, a value of *p* ≤0.05 was considered statistically significant.

## Results

### Description of enrolled population

**Figure 1:**
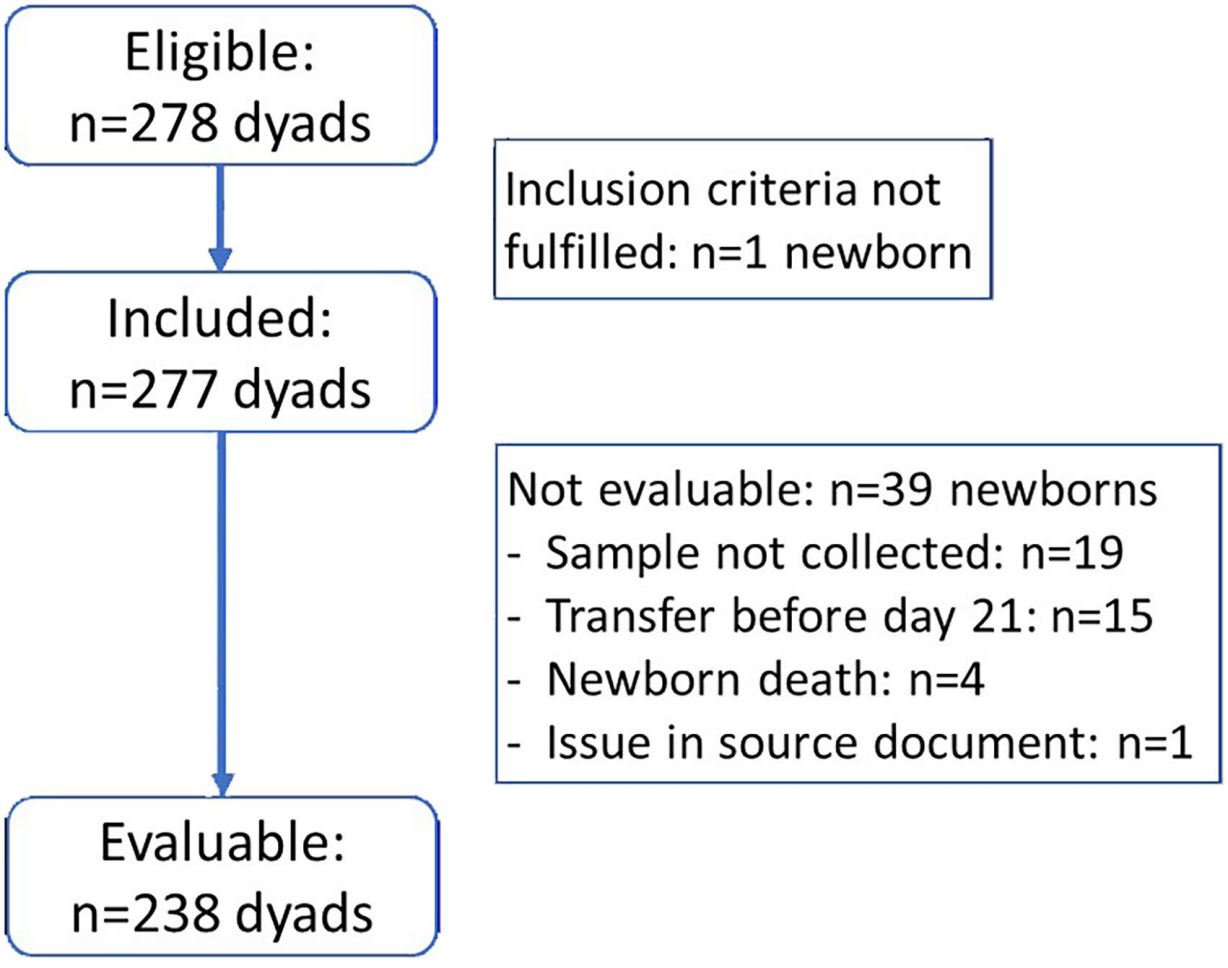
Study participants flowchart.

A total of 278 preterm newborns were included in this study, leading to 238 evaluable dyads (238 evaluable newborns and 194 mothers) (Figure 1; Table 1). Multiple gestations accounted for 21.6% dyads, with 40 pairs of twins and 2 sets of triplets (36.1% newborns). Births resulted mostly from cesarean section (cs) (62.6%).

**Table 1:** Study participants characteristics (mothers and newborns, in number of dyads).

Variables [*n* (%) or mean (SD)]	Dyads (*n* = 238)	
Mothers		
Age (year)	31.0 (5.7)	
During pregnancy		
Infection: yes	30	12.6%
Not aware	22	9.2%
Antibiotics	44	18.5%
During delivery		
Antibiotics	84	35.3%
Fever	4	1.7%
Premature rupture of membranes	85	35.7%
Duration (hours)	10 [0.1;48]	
Type of delivery		
Vaginal birth	89	37.4%
Cesarean section	149	62.6%
Factors of pre-term birth		
Vascular disorders	58	24.4%
Intra-uterine growth restrictions	64	26.9%
Chorioamnionitis	15	6.30%
Multiple gestation	82	34.5%
Premature delivery threats	120	50.4%
Other	44	18.5%
Several factors combined	129	54.2%
Samples		
Vaginal sample collected	46	19.3%
Feces sample collected	209	87.8%
Newborns		
Female sex	136	57.1%
Gestational age (weeks)	31.3 (1.7)	
Weight at birth (g)	1,446 (368)	
Weight at discharge (g)	2,304 (396)	
Length of hospital stay (days)	39.5 (15.9)	
Feeding of newborns		
Mother’s milk* as first intake	189	79.4%
Mother’s milk* at J7	186	78.2%
Mother’s milk* at J21	145	60.9%
Mother’s milk* at discharge	102	42.9%
Probiotics	21	8.8%
Infectious episode in newborns		
At least one episode	86	36.1%
Associated maternal-fetal infection	20	8.4%
Suspected episode	58	24.4%
Confirmed with bacterial strain isolated	32	13.4%
GNB as causative pathogen	10	4.2%
With acquired resistance	4	1.7%
Producing ESBL and/or Hcase	0	0.0%
Resistance integrons detected	1	0.4%
Antibiotic treatment administered	78	32.8%
For at least 48 h	42	17.6%

Hcase, hyperproduction of cephalosporinase D; ESBL, extended spectrum beta-lactamase; GNB, Gram-negative bacteria; IQR, interquartile range. *mother milk was either the raw or pasteurized breast milk of the newborn’s mother.

The main characteristics of mothers and newborns, and of delivery, are shown in Table 1. During pregnancy, antibiotics were administered to 21.3% dyads (Supplementary Figure S1A), while, during delivery, antibiotics were administered to 50.3% dyads (Supplementary Figure S1B), primarily in case of premature rupture of membranes (72.6%). Overall, 7.3% dyads received antibiotics both during pregnancy and delivery.

While most newborns remained hospitalized until day 21 (98.7%), 44.5% were still hospitalized at day 36, 16.0% at day 51, and 2.5% at day 66. They were 81.9% discharges to home, 17.6% referrals to community centers, and 1.7% deaths at hospital. Details on newborns feeding from day 1 to day 21 are in Supplementary Figure S2. Overall, 47.4% newborns received the same type of nutrition from birth to day 7, which was pursued until day 21 for 26.5% of them.

### Prevalence of GNB, MDR-GNB, and integrons in mothers

Among the vaginal samples performed in 19.3% dyads, 32.6% samples yielded 25 GNB overall. The isolated GNB were almost only *Enterobacterales* (96.0%), mostly *Escherichia coli* (Table 2). Four MDR-GNB isolates (Table 3) were isolated in 20.0% out of the 15 dyads with GNB. Integrons were detected in the ESBL *E. coli* strain.

**Table 2:** Description of gram-negative bacteria isolated in samples from mothers and newborns.

Bacterial characteristics	Maternal vaginal fluid	Maternal feces	Newborn feces
Susceptibility profile	(*n* = 25 strains)	(*n* = 384 strains)	(*n* = 893 strains)
Gram-negative bacteria without any acquired drug resistance	17 (68%)	265 (69%)	678 (76%)
Only multi-drug-resistant Gram-negative bacteria	4 (16%)	24 (6%)	72 (8%)
Only other type of acquired resistance	4 (16%)	89 (23%)	138 (15%)
Both types of acquired resistance	–	6 (2%)	5 (1%)
Bacterial species	(*n* = 25 strains)	(*n* = 384 strains)	(*n* = 898 strains)
*Escherichia coli*	13 (52%)	238 (62%)	320 (36%)
Other enterobacteria	11 (44%)	79 (21%)	529 (59%)
*Pseudomonas* spp.	–	36 (9%)	28 (3%)
Other non-fermenting Gram-negative bacteria	1 (4%)	31 (8%)	4 (2%)

**Table 3:** Bacterial species of multi-resistant strains of Gram-negative bacteria isolated in maternal and newborns’ samples.

Samples from which multi-resistant strains were isolated:	Extended spectrum beta-lactamase-producing strains (ESBL)	Strains with hyperproduction of cephalosporinase (Hcase)
Maternal samples		
Vaginal samples	ESBL alone: *n* = 1	Hcase alone: *n* = 3
Feces	ESBL alone: *n* = 8 ESBL combined with other acquired resistance: *n* = 5	Hcase alone: *n* = 16 Hcase combined with other acquired resistance: *n* = 1
Newborn feces	ESBL alone: *n* = 6 ESBL combined with other acquired resistance: *n* = 6	Hcase alone: *n* = 66 Hcase combined with other acquired resistance: *n* = 0

Maternal feces were collected in 87.8% dyads, and 96.2% samples yielded 384 GNB strains. The isolated GNB were mostly *Enterobacterales* (82.5%; Table 2). Acquired resistance to at least one antibiotic was identified in 31.0% strains, with 25.2% MDR-GNB strains (including 43% ESBL and 57% Hcase), and 74.8% strains with solely other type of acquired resistance (Table 3).

Integron were detected in 43.1% feces and 10.4% GNB, including class 1 in 36.5% feces and 9.0% GNB, class 2 in 11.1% feces and 1.6% GNB, and class 3 in 4.8% feces and none of GNB. Overall, 74% of strains without any resistance phenotype did not harbor any integron, and 24.1% of strains with acquired resistance (ESBL, Hcase or other acquired resistance) harbored at least one integron, classes 1 or 2 (Chi2 test, *p* < 0.0001). Four strains were both ESBL-producing and with a class 1 integron.

For the mothers, the negative predictive value (NPV) of integrons detection in GNB isolated from feces was 96% for 3CG third generation cephalosproins, 97.8% for SXT and 88% for aminoglycosides.

### Acquisition of GNB, MDR-GBN, and integrons in feces of preterm newborns

A median [IQR] of 4 [4; 5] samples of feces were collected by newborn at predefined timepoints during their stay, with at least 3 fecal samples by newborn.

**Figure 2:**
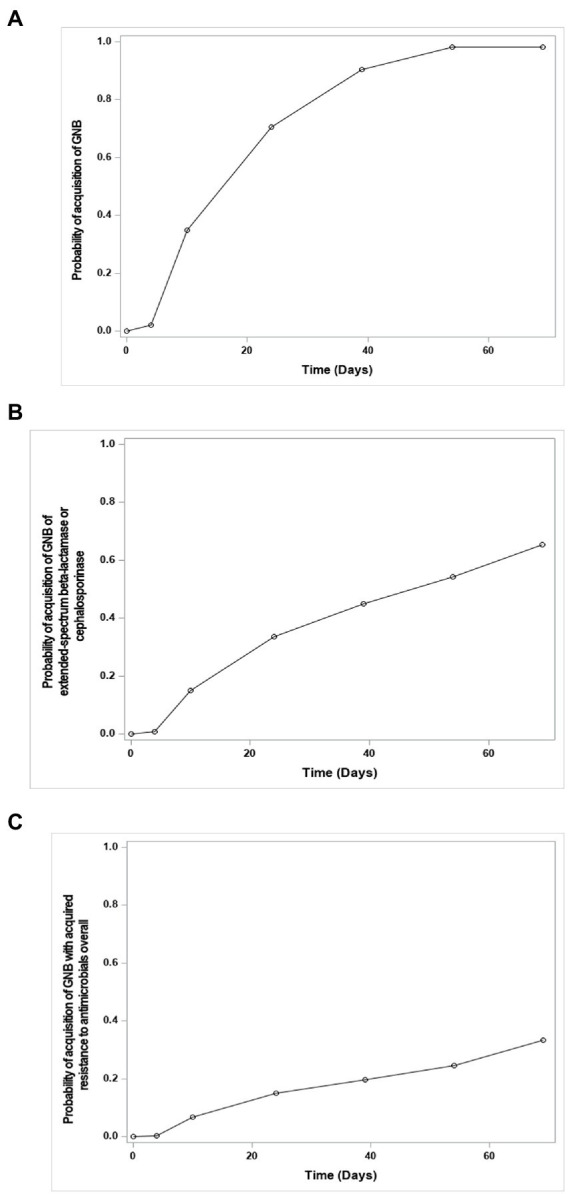
Curves of the acquisition over time by newborns of Gram-negative bacteria (GNB) (curve **A**), of multidrug-resistant Gram-negative bacteria (MDR-GNB) (curve **B**), or of GNB with other acquired resistance to antimicrobials overall (curve **C**) in feces of newborns. More specifically, the curves show the inverse of the survival probability of the non-acquisition of the studied event over time, estimated by the actuarial method of survival analyzes.

The GNB colonization of newborns was progressive, from 8.8% colonized in their first sample to 88.7% colonized at discharge (Figure 2). Among these 211 newborns, 103 became carriers of GNB with any type of resistance to antibiotics, including 26 newborns with solely MDR-GNB, 59 with solely other acquired resistance, and 18 with both. Factors identified by multivariate analysis as independently associated with the acquisition of GNB are presented in Table 4. Overall, 898 GNB strains were isolated from newborns, and susceptibility profile to antibiotics run for 893. The majority (75.9%) of the strains had no acquired resistance; strains with acquired resistance are described in Table 3.

**Table 4:** Factors related to the mother, the type of delivery of the newborn and newborns’ characteristics, that were identified as independently involved in the acquisition by newborns of Gram-negative bacteria (GNB), multidrug-resistant Gram-negative bacteria (MDR-GNB), and integrons.

Independently associated variables for each type of acquisition	Hazard ratio	95% confidence interval	*p* value
Acquisition of GNB			
Gestational age (by additional week of amenorrhea)	2.04	1.45; 2.86	<0.0001
Multiple gestation	2.04	1.45; 2.86	<0.0001
Prematurity due to Intra-uterine growth restrictions	0.64	0.44; 0.93	0.02
Vaginal delivery	1.99	1.38; 2.86	0.0002
Artificial milk during hospital stay	0.60	0.42; 0.86	0.006
Acquisition of MDR-GNB			
Prematurity due to premature rupture of membranes	3.41	1.71; 6.81	0.0005
Acquisition of GNB with other acquired resistance			
Antibiotics during delivery	2.34	1.48; 3.69	0.0003
Maternal milk during hospital stay	1.65	1.05; 2.59	0.03
Acquisition of integrons			
Multiple gestation	0.38	0.19; 0.73	0.02
Artificial milk during hospital stay	0.54	0.32;0.90	0.004
Carriage of integrons in mother’ feces	2.00	1.18; 3.38	0.01

**Figure 3:**
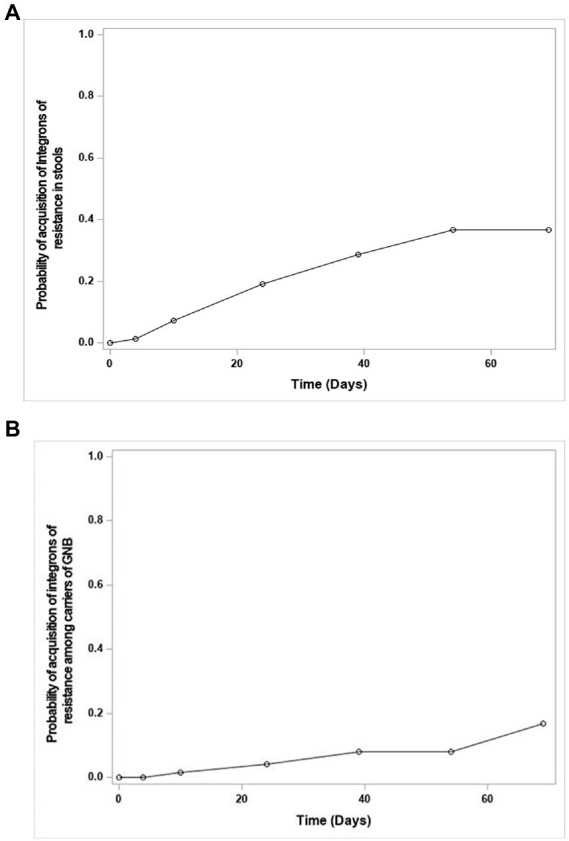
Curves of the probability of acquisition over time of integrons in feces of newborns; among all newborns (Curve **A**); among newborns carriers of Gram-negative bacteria (GNB; Curve **B**). More specifically, the curves show the inverse of the survival probability of the non-acquisition of the studied event over time, estimated by the actuarial method of survival analyzes.

The number of newborns with detected integrons increased progressively over time from 3.0% of the 231 evaluable samples to 27.6% (Figure 3A). Among newborns who were GNB carriers (*n* = 211), integrons were detected in 8.5%, of whom 15 had an integron-positive GNB, also with a progressive acquisition of integrons over time (Figure 3B). Factors identified by multivariate analysis as independently associated with the acquisition of integrons are presented in Table 4.

Integrons were investigated in 875 strains of GNB; 3.2% had at least one integron (class 1: 2.9%, classes 1, and 2: 0.3%). The 3 strains with the combination of classes 1 and 2 harbored an acquired resistance to beta-lactams and quinolones. Overall, 10.7% of the strains with integrons expressed ESBL or Hcase, 57.1% expressed another type of acquired resistance, while 77.6% of the strains without any resistance phenotype did not harbor any integron (Chi2 test, *p* < 0.0001).

Among integron-positive newborns, integrons were also detected in 50.8% maternal samples, while 55.4% of the negative newborns had also no integron detected in maternal samples (Chi2 test, *p* = 0.024).

The NPV of integron detection in GNB isolated from newborns’ feces was 94.5% for resistance to 3GC, 96.8% for SXT and 94.5% for aminoglycosides.

Strains of identical species were identified in both mother and newborns for 89 dyads, of which 61% were delivered by vaginal delivery (vd) and 39% by cs. Strains were RAPD-identical between mother and newborn in 38.2% dyads (vd, 44.4% and cs, 28.6%), mostly with an *E. coli* strain (94.1%), otherwise a *Klebsiella pneumoniae* strain (5.9%). Three dyads had strains of identical species but different susceptibility profiles. Of note, most siblings (80.9%) had consistent integrons carriage.

## Discussion

We present here a multicenter study conducted in both mothers and preterm infants, a total of 238 dyads. The aim of the study was to investigate the acquisition of GNB, resistant or not, and integrons by newborns. In mothers, carriage of cultivable GNB and the presence of integrons were examined in two body sites, vaginal fluids and feces, to provide a more complete picture. To our knowledge, this is one of the first studies conducted on such a large number of preterm infants in association with their mothers. The main causes of preterm birth were hypertensive disorders, fetal growth restriction, preterm labor with or without premature rupture of membranes, and multiple gestation. The rate of cs (62%) was similar to that observed in the French epidemiological study Epipage 2 for similar gestational ages (Ancel et al., 2015), and, as expected, almost 3-times higher than that observed in our study in term newborns (22.1%; Barraud et al., 2018). This observation is consistent with previous studies that have consistently shown a higher rate of cesarean delivery in preterm infants compared with term infants (Collado et al., 2015). In addition, antibiotic prophylaxis is recommended in case of premature rupture of membranes to avoid possible infection of the preterm infant. This could explain the higher rate of antibiotic administration during delivery in our study (35.4%) compared to the previous one (25.4%).

### Acquisition of GNB and MDR-GBN in feces of preterm newborns

As in other studies (Jiménez et al., 2008; Barraud et al., 2018), almost all newborns were progressively colonized with GNB during their hospital stay, with *E. coli* being the first and most frequent Gram-negative colonizer. However, the rate of newborns carrying GNB in their first fecal sample was much lower in our study, 8.8%, compared with 22.7% in the previous study (Barraud et al., 2018). The latter study was conducted in term newborns, whereas our study focused on preterm newborns born at a mean gestational age of 31 weeks. This lower gestational age also implies a shorter exposure to maternal GNB colonization during pregnancy, as already described by Walker *et al* (Walker et al., 2017). This is consistent with the association between increased acquisition of GNB and increased gestational age observed in this study. In fact, this colonization was independently favored by higher gestational age, multiple gestation, or vaginal delivery, and decreased in the case of prematurity due to intrauterine growth restriction or feeding with artificial milk during the neonatal hospitalization. However, as in the study in term infants, no association was identified between GNB acquisition and antibiotic exposure during delivery. Importantly, the administration of antibiotics to newborns during the first weeks of life was also not significantly associated with the acquisition of GNB, resistant or not, or with the detection of integrons. The decrease in GNB acquisition with formula feeding is consistent with maternal gut microbiota reaching the breast milk to influence neonatal gut colonization (Jost et al., 2014).

At discharge, nearly half (43%) of the preterm infants were colonized with resistant GNB. Of note, strains of GNB with acquired resistance to at least one antibiotic were isolated from the feces of only 31% of mothers. The gap between the two colonization rates is likely explained by a probable hospital origin of the neonatal acquisition of resistant GNB. Of note, we did not correlate the carriage of MDR-GNB in mothers with that in newborns. When an identical GNB strain was found in both mother and preterm newborn, vaginal delivery predominated (24 vd versus 10 cs).

Regarding MDR-GNB, we observed a low rate of colonization, as previously described by our team (Barraud et al., 2018). It should be noted that studies from other teams are difficult to compare because of the different methods used. Premature rupture of membranes was an independent risk factor for MDR-GNB, whereas antibiotic administration during delivery or breastfeeding during hospitalization favored the acquisition of GNB with other types of acquired resistance.

Although only 3 newborns had markers of multidrug resistance (resistance to at least 3 antibiotic families) in their first postnatal feces, it shows that acquisition of resistant GNB likely begins before birth, consistent with descriptions by other teams (Jiménez et al., 2008).

### Acquisition of integrons in feces of preterm newborns

Integrons were searched for directly in meconium and feces samples and in GNB isolates. The presence of integrons was detected in one out of four newborns. It progressively increased, as for GNB acquisition. As for resistant GNB, the acquisition of integrons likely begins before birth, as 7 (2.9%) newborns had integrons in their first post-natal feces. This is consistent with previous descriptions of pre-birth colonization of the gut microbiota (Basu, 2015). Our findings on the presence of integrons in maternal feces (40%) are also consistent with that of another study conducted in the general population (42%) (Chainier et al., 2017). Integron presence in preterm and term newborns is less frequent than in mothers, confirming that integron acquisition occurs throughout life. Progressive acquisition of integron-carrying strains in preterm infants was independently associated with the colonization of maternal feces with integron-carrying strains, whereas multiple gestation or formula feeding were independent protective factors. This last point is probably explained by the lower contamination of artificial milk compared to human milk, even when pasteurized. Surprisingly, antibiotic administration during delivery was not a risk factor for acquisition of integron-carrying strains in preterm infants, in contrast to term infants (Barraud et al., 2018). This may be related to the high frequency of antibiotic administration in preterm infants. In both populations of our study, class 1 integrons were the most prevalent, followed by class 2 integrons, as previously described (Chainier et al., 2017; Barraud et al., 2018). Of note, class 3 integrons were detected in 4 maternal feces, which is quite uncommon in human feces (Chainier et al., 2017). In GNB strains, integrons were detected mostly in *E. coli* strains. As shown in previous studies (Chainier et al., 2017; Barraud et al., 2018), integrons were detected more frequently in feces than in bacteria, possibly because (i) presence of integrons in non-cultivable bacteria, (ii) some bacteria may not have been collected during the culture process, and (iii) false-negative cultures due to antibiotic treatment. Finally, in our study we showed a good NPV of integrons detection in both preterm infants and mothers for the resistance to 3GC, SXT, and only in preterm infants for aminoglycosides resistance, as described in some studies (Barraud et al., 2021; Elias et al., 2022). These results suggest that integrons could be used as predictive markers for the absence of antibiotic resistance in fecal samples from various adult and neonatal populations.

### Study limitations

However, our study has limitations. First, newborns were followed until hospital discharge, thus for a variable duration. However, the risk of acquiring resistant pathogens is associated with hospitalization. Second, we studied only cultivable GNB, which may not be sufficient to adequately track GNB acquisition. Third, the study was conducted in France, and thus may reflect national specificities, e.g., for antibiotic resistance epidemiology, for decisions on antibiotic administration or for pregnancy- or childbirth-related practices. Finally, studies conducted by other teams with similar goals used different investigation methodologies, such as new generation sequencing, making it difficult to compare the results.

### Conclusion

This large multicenter study, which investigated the dynamics of the acquisition of GNB, resistant or not, and GNB with integrons in preterm infants, in the light of the maternal colonization in feces and vaginal fluids, showed that neonatal acquisitions of GNB and of GNB with integrons were progressive. The main independent risk factor for the acquisition of MDR-GNB was premature rupture of membranes, while the main independent protective factors for the acquisition of GNB with other acquired resistance mechanisms were the antibiotics administration during the delivery, and the feeding of the newborns with breast milk during hospitalization. No specific risk factors were identified for the acquisition of strains with integrons, which was only associated with the colonization of maternal feces by integron-carrying strains. The progressive colonization of newborns suggests the involvement of the microbiological ecology of the neonatal unit and neonatal management, which warrants further studies.

## Data availability statement

The raw data supporting the conclusions of this article will be made available by the authors, without undue reservation.

## Ethics statement

The studies involving human participants were reviewed and approved by Ethic Committee of Limoges (Comité pour la Protection des Personnes du Sud-Ouest et Outremer #4), Approval number #CPP14-017/2014-A00283-44 dated 18 March 2014. Written informed consent to participate in this study was provided by the participants’ legal guardian/next of kin.

## Author contributions

AL analyzed the data. SL recovered and analyzed the data. PB, LR, MB, LP, KN, and PJ included patients. AP, PL, DD, SM, LV, and DL performed analyses and recovered data. DC and CG recovered and analyzed data. M-CP proofread the manuscript. AB included patients and proofread the manuscript. FG performed analyses, analyzed data, wrote, and proofread the manuscript.

## Funding

This work was supported by a grant from the French Health Ministry [Ministère des Affaires Sociales et de la Santé, Direction Générale de l’Offre de Soins (DGOS)].

## Conflict of interest

The authors declare that the research was conducted in the absence of any commercial or financial relationships that could be construed as a potential conflict of interest.

## Publisher’s note

All claims expressed in this article are solely those of the authors and do not necessarily represent those of their affiliated organizations, or those of the publisher, the editors and the reviewers. Any product that may be evaluated in this article, or claim that may be made by its manufacturer, is not guaranteed or endorsed by the publisher.
