# Supplementary material for: Dynamics of the digestive acquisition of bacterial carriage and integron presence by French preterm newborns according to maternal colonization: The DAIR3N multicentric study
Source: Front Microbiol. 2023 Mar 14;14:1148319. doi: 10.3389/fmicb.2023.1148319 (PMC10043237; doi:10.3389/fmicb.2023.1148319)

Supplementary Material

Dynamics of bacterial carriage and integron presence in gut of French preterm new-borns according to maternal colonization: the DAIR3N multicentric study

[1]A. Patry, [2]Bothorel P., [3]A. Labrunie, [4]L. Renesme, [5]P. Lehours, [6]M. Benard, [7]D. Dubois, [2]L. Ponthier, [1]S. Meyer, [8]K. Norbert, [9]L. Villeneuve, [10]P. Jouvencel, [11]D. Leysenne, [1]D. Chainier, [3]S. Luce, [1]C. Grélaud, [2]M.C. Ploy, [2]A. Bedu, [1]*F Garnier

*** Correspondence:** Corresponding Author: Fabien.GARNIER@unilim.fr

Supplementary Figures

e-Figure 1 - Antibiotics administered (A) during pregnancy (n=44/206 dyads, combination of antibiotics, n=5 dyads, missing data, n=32 dyads); (B) during the delivery (n=84/167 dyads, combination of antibiotics, n=8 dyads, missing data n=71 dyads)

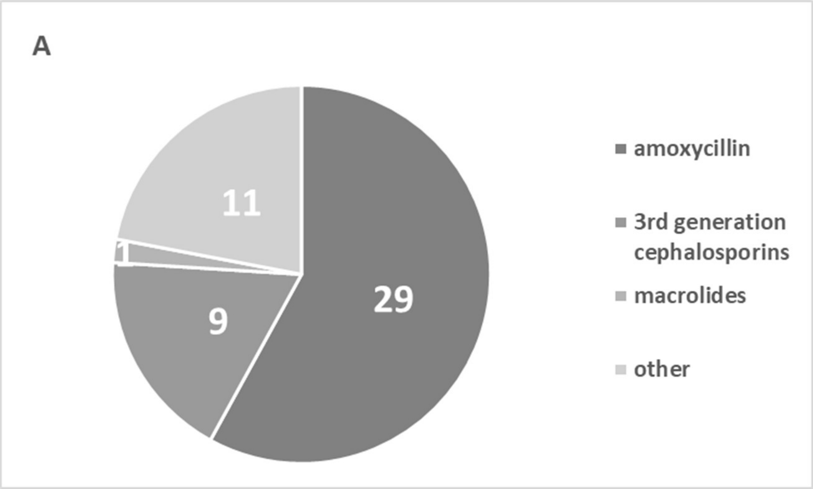

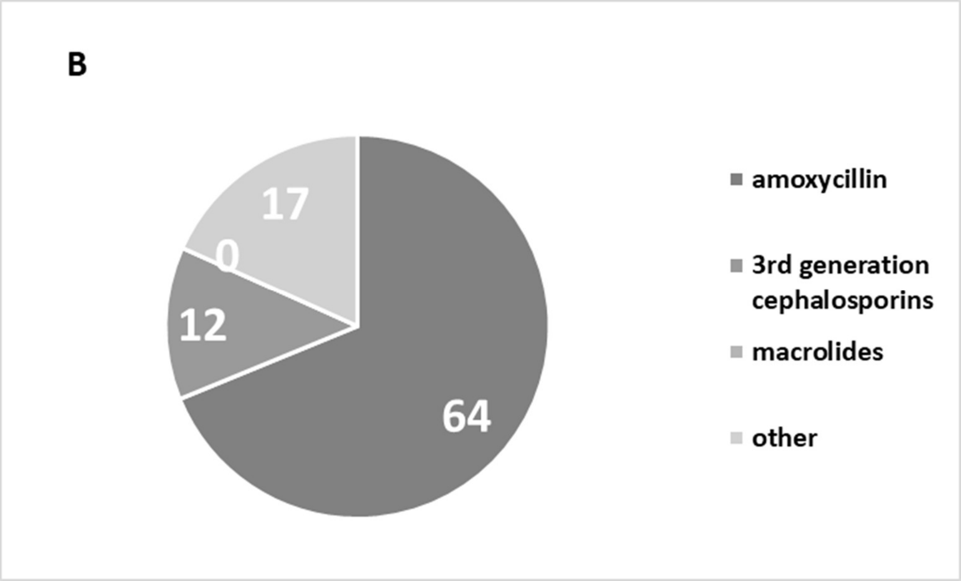

e-Figure 2 – Type of nutrition received by newborns from birth (day 1) to day 21 (n=238 newborns)

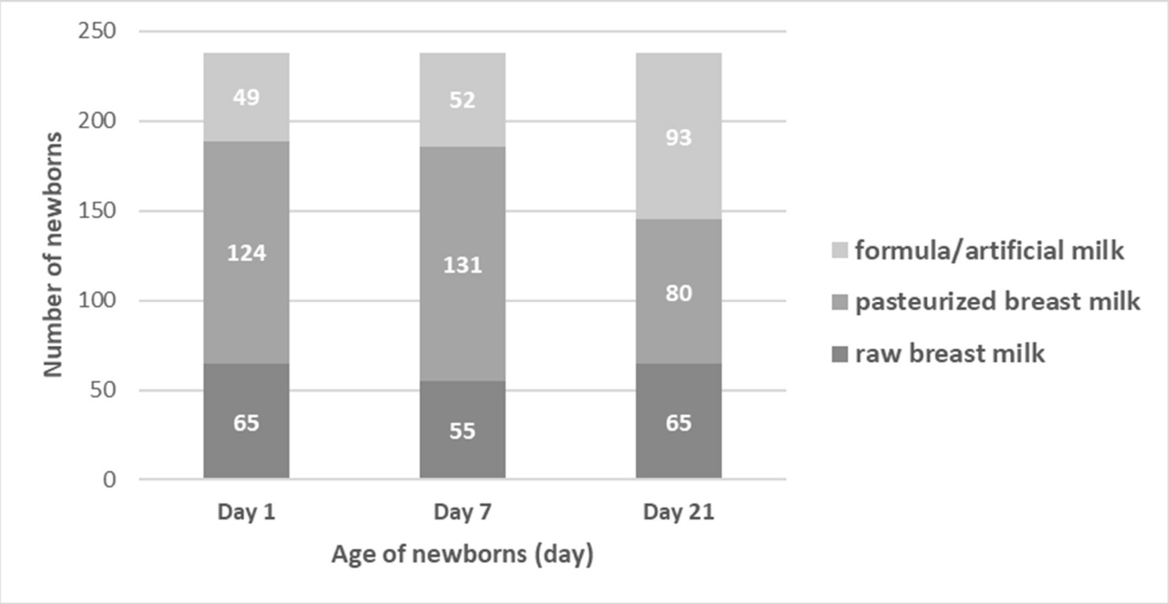

Supplement: Supplementary file 1 [file Data_Sheet_1.PDF]
